# Supplementary figures and images for: The bloodstream form of Trypanosoma brucei displays non-canonical gluconeogenesis
Source: PLoS Negl Trop Dis. 2024 Feb 23;18(2):e0012007. doi: 10.1371/journal.pntd.0012007 (PMC10917290; doi:10.1371/journal.pntd.0012007)

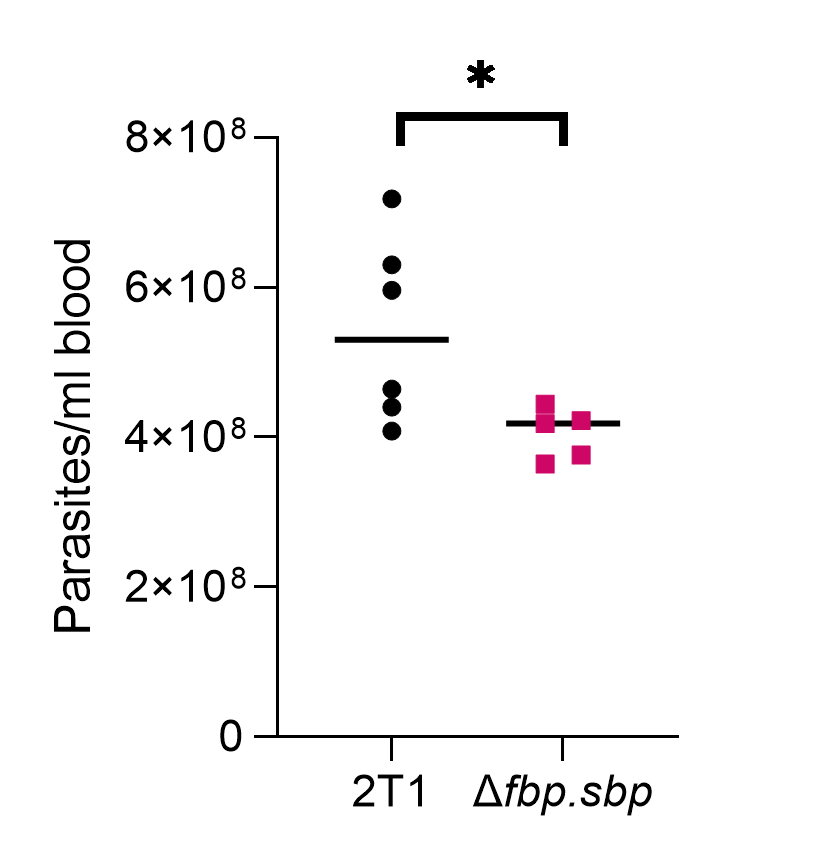

Supplement: S1 Fig — (TIF) [file pntd.0012007.s001.tif]

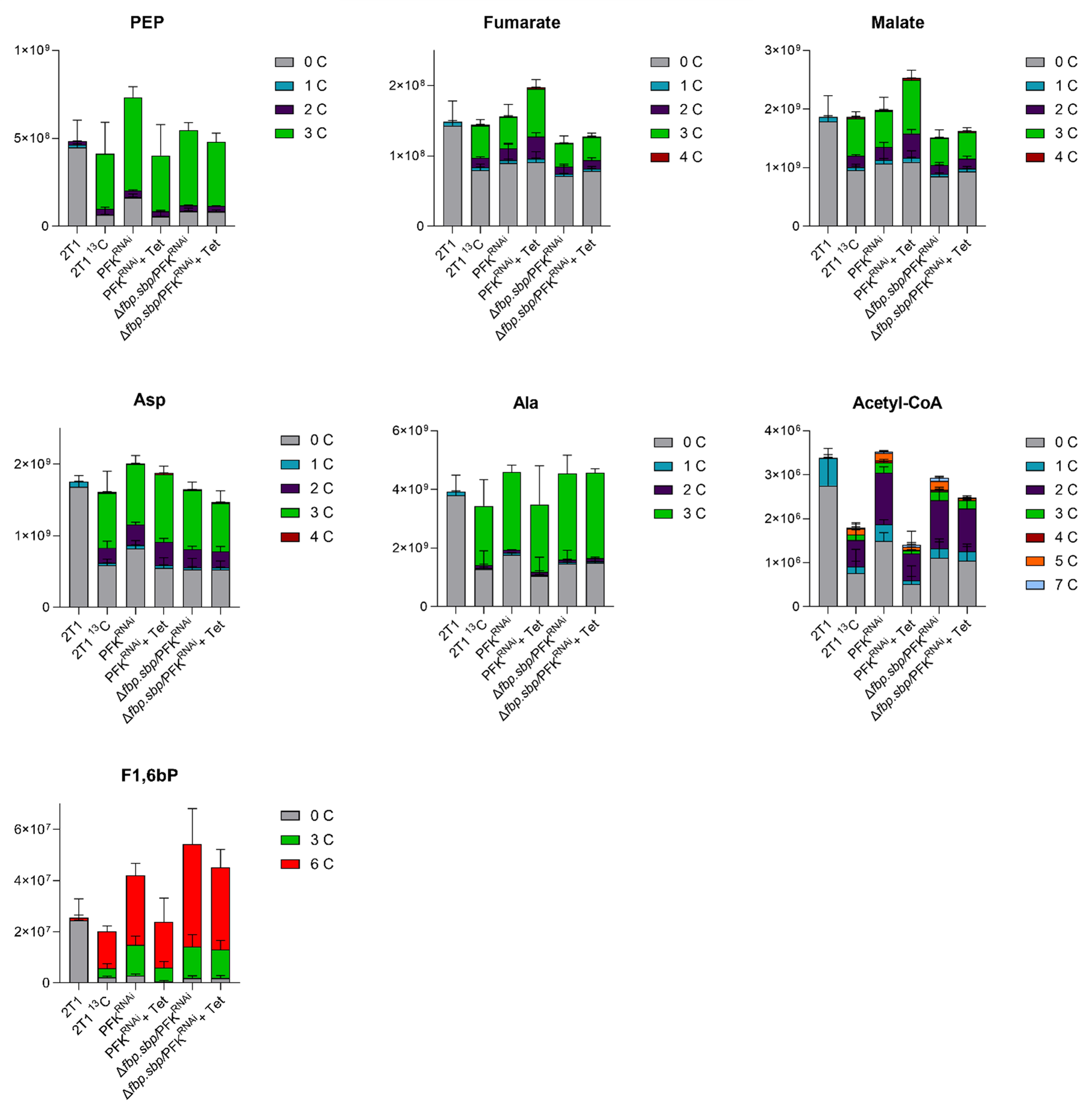

Supplement: S2 Fig — 2T1 13C –parental cell line in medium with 13C-glycerol, PFKRNAi–non-induced PFK RNAi cell lines in medium with 13C-glycerol, PFKRNAi + Tet–PFK RNAi induced for 24 h in medium with 13C-glycerol, Δfbp.sbp/PFK–non-induced Δfbp.sbp/RNAiPFK cell line in medium with 13C-glycerol, Δfbp.sbp/PFK + Tet—Δfbp.sbp/RNAiPFK cell line induced for 24 h in medium with 13C-glycerol. (TIF) [file pntd.0012007.s002.tif]

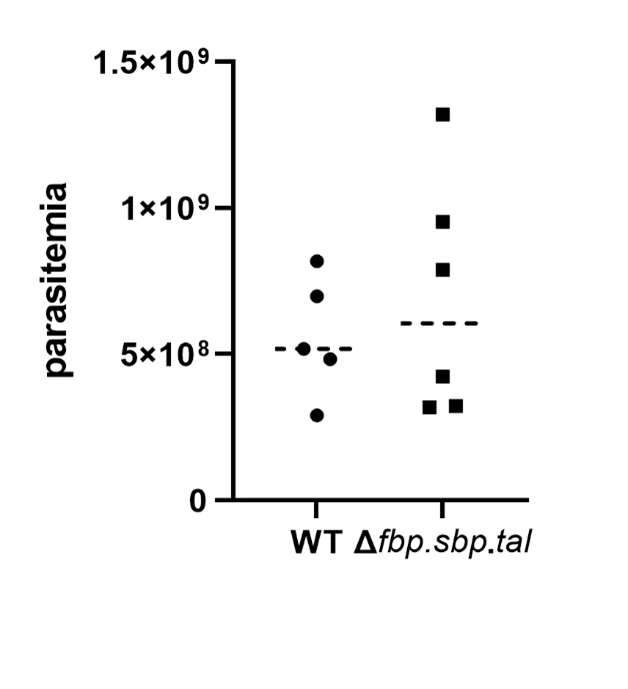

Supplement: S3 Fig — (TIF) [file pntd.0012007.s003.tif]

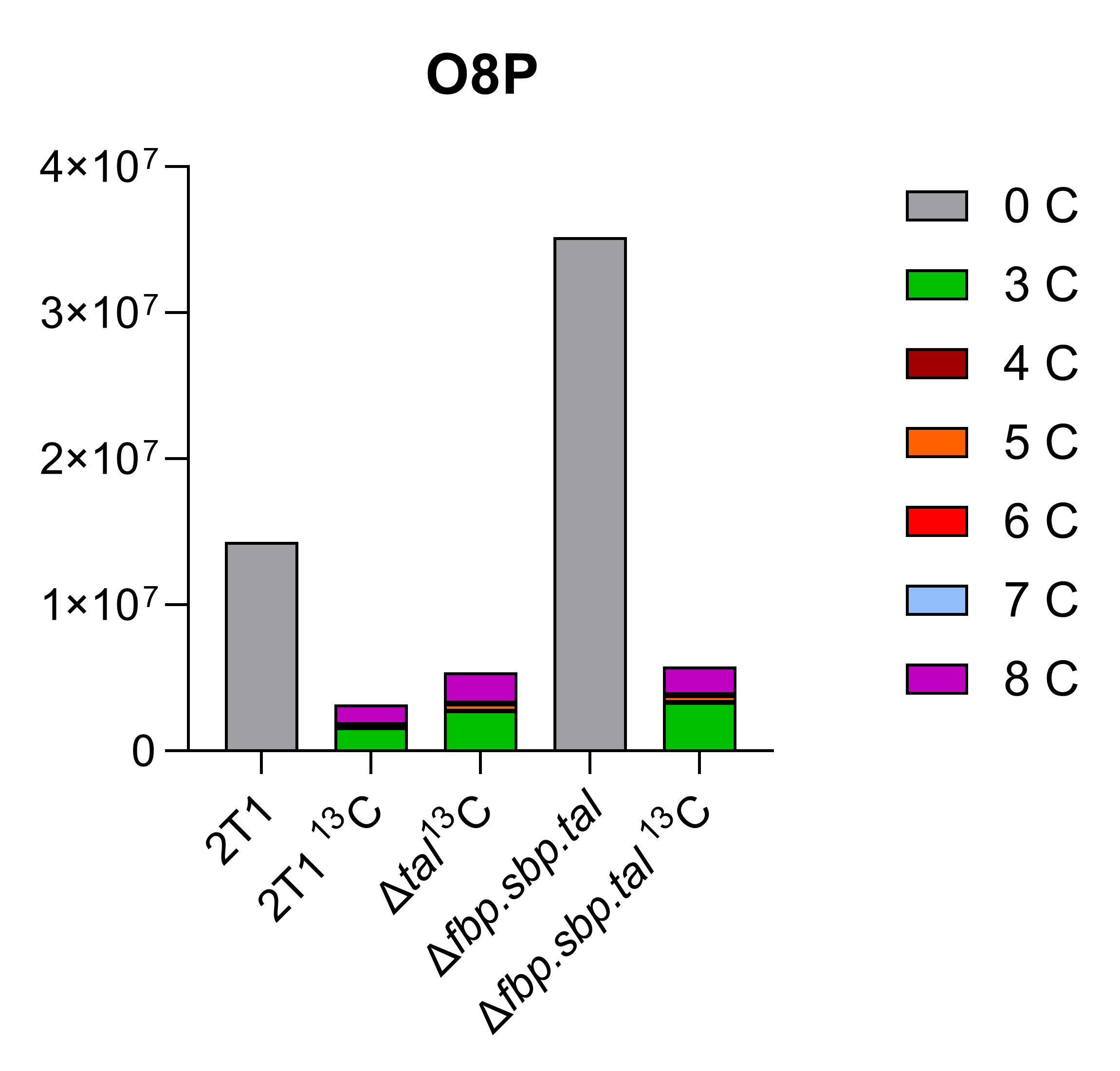

Supplement: S4 Fig — 13C indicates medium supplemented with 13C-glycerol as the only carbon source. (TIF) [file pntd.0012007.s004.tif]
