# Supplementary material for: The bloodstream form of Trypanosoma brucei displays non-canonical gluconeogenesis
Source: PLoS Negl Trop Dis. 2024 Feb 23;18(2):e0012007. doi: 10.1371/journal.pntd.0012007 (PMC10917290; doi:10.1371/journal.pntd.0012007)
Supplement: Attachment — Submitted filename: Response to reviewers.docx [file pntd.0012007.s007.docx]

*January 20, 2024*

*Dear Editor, Dear Reviewers,*

*We would like to thank you for your time that you dedicated to this work. We are grateful for your comments and suggestions that helped to improve the manuscript.*

*We added the metabolomic experiment on double KO as required, and provided Figures with markers (Fig 1), an additional PCR (Fig 5), and supplied standards for the metabolomic experiments (S1 Table).*

Major concerns:
1.      Parameters for metabolomics data processing should be provided, with particular emphasis on methods used to provide and confirm metabolite annotations.

*As mentioned in the Methods, metabolites were annotated based on matches with respective standards. Histograms for both the standards and the metabolites shown in Figures throughout the manuscript were added to S1 Table, Sheet 2.*

2.      Multiple lines in the manuscript refer to targeted metabolomics (eg 403, 491), yet the methods section only describes untargeted metabolomics approaches. If targeted approaches were performed (for example, data acquisition in PRM mode), then the inclusion list should be provided.

*By “targeted metabolomics” we referred to the use of labelled substrate ^13^C_3_-glycerol. We have now replaced this wording in the manuscript.*

3.      Given the surprising findings of lack of effect of the KO on 13C incorporation, data supporting the annotations of the metabolites displayed in Fig 3, Fig 5, Fig S2 should be provided, such as retention time matching to the standards run by the authors.

*As mentioned above, histograms for both the standards and the metabolites have been added to S1 Table.*

4.      The growth defect of the Δfbp.sbp is most pronounced in CMM media, and the untargeted metabolomic changes reported in Fig 2AB are likewise from growth in CMM media. It is therefore critical that the 13C tracing experiments for the double KO strain also be performed in CMM media. That would also enable comparison with the triple KO in Figure 5D.

*The experiment of* Δfbp.sbp *cells grown in CMM with ^13^C_3_-glycerol was performed and the data supplied to Figure 2D. The major changes are actually observed due to replacement of glucose with glycerol. For instance, profile for G6P is similar for the double and triple KO strains, since the levels decrease in glycerol medium substantially.*

5.      S1 figure: if available, adding additional data covering multiple acute-stage timepoints or additional replicates would provide stronger confirmation that the KO strains do indeed present with a growth defect. The fact that the triple KO strain in Figure S3 showed no phenotype suggests the possibility that the findings in Figure S1 might not be reproducible (especially since the magnitude of the phenotype was so small).

*We are aware that the change is rather small and the p-value high, hence we did not draw any major conclusions from this observation. Regarding the discrepancy with the triple KO, it is possible that the cells underwent an adaptation during the process of the triple KO generation, which would make them more resistant to metabolic stress and could explain why we observed a difference in the former experiment with the double KO, but not later with the triple KO. However, we do not consider it ethical to repeat the infectivity experiment, especially considering the metabolomic results showing persistance of GNG flux.*

6.      Based on the primers provided in Table 1, the product confirming SBP deletion should be 612 bp, but in Fig 1C, it’s marked at 700 bp. The full gel with full ladder should be provided for both panels in Fig 1C, to confirm that products are at the expected size.

*The ladder was included in the panels in Fig 1. The details regarding oligos annealing in Table 1 were also corrected, since the oligos AZ1211 and AZ 1212 amplify bases 93 – 813 of FBP ORF resulting in a product of 720 bp. For SBP, the oligos amplify an ORF of 276 – 888 bp, the size of 612 bp.*

*The full gel images are provided below.*

7.      Table 1 indicates that primers were also used to confirm replacement with the drug resistance cassette (not just loss of the internal primer amplification product). That data is not provided in the manuscript but should be added.

*The primers listed in Table 1 were actually used for amplification of the repair cassettes used for replacement of the respective ORFs by Cas9 editing. Nevertheless, we included this data for generation of the ∆tal cell lines, where validation by a western blot is not available. The new data is included in Figure 5, and the primers used added in Table 1. These are primers annealing to the UTRs of the TAL gene, hence amplifying the TAL ORF in the parental cell line, and the puromycin and phleomycin resistance cassettes in the KO cell lines.*

Minor concerns:
1.      I would recommend that authors deposit their metabolomics data in a public data repository.

*All the raw data is publicly available form the Figshare database.*

2.      Clone numbers should be added to Figure 4A, so that readers can confirm that the clones with the correct Western blot band in Figure 4B match with the clones with the correct PCR band in Figure 4A.

*The clone numbers were added.*

3.      Did the triple KO strain show a growth defect in CMM media? Such data would provide an interesting complement to the in vivo data and to the growth data for the double KO, but is not mandatory.

*The triple KO did not show a significant growth defect in the CMM media, as shown below. As mentioned above, the cells probably underwent a metabolic adaptation during the process of generation of triple KO.*

Reviewer #2

In my opinion, despite this argument is common, it is weak since the activity depends on the thermodynamics conditions of the reaction and (of course) on the relative quantities of substrate and products. According to Equilibrator (https://equilibrator.weizmann.ac.il/) at neutra pH the reaction: ATP(aq) + D-Fructose-6-phosphate(aq) <=> ADP(aq) + D-Fructose-1,6-bisphosphate(aq) would have an equilibrium constant of ~3.6 e2. This could be reversible in physiological conditions, considering a situation of virtual absence of F6P and low ATP (see my other comment below)

*We adjusted our statement to address the reviewer comment (lines 126 – 131). It now reads as follows:*

*Fernandes and colleagues [17] reported reverse activity of trypanosomal and mammalian PFKs in vitro, but the physiological relevance is unclear. The reverse activity of three mammalian PFK isoforms is not thought to occur in vivo [18] and although trypanosomal PFK is localised in specialized organelles called glycosomes, which could theoretically form an environment that allows reverse activity, experimental evidence is still lacking.*

I think this should not be a statement. Maybe better to say that it is expectable that the absence of FBPase does not have such a substantial impact on their abundance. Even more, as the metabolomics assay does not measure absolute quantities, the correct expression should be that relative quantities remains unchanged (here and all over the text). Or this assay setup allows to rule out that maybe both metabolites changed together?

*We are showing amounts of each metabolite relative to the parental cell line, so we know that both of these metabolites did not change together. We adjusted our statement to address the reviewer comment (lines 342 – 344). It now reads as follows:*

*Considering that F6P and F1,6bP are also involved in other metabolic pathways, particularly glycolysis, the absence of FBPase is not expected to have such a large impact on their relative abundance.*

All this paragraph describes a relevant experiment, supporting the idea that PFK is not responsible for complementing the fbp knock out. However, it does not completely rule out this possibility. The authors show that iRNA-depleted PFK lineages proliferate less. iRNA diminishes PFK expression by 20% (maybe a similar percent taking into account the slopes of the curves?). Therefore, it should be considered the possibility that the diminished proliferation could be a compensation for the diminished both, direct and reverse reaction of PFK. I recognize that the authors´ hypothesis looks very likely, but other possibilities cannot be excluded and should be discussed.

*We agree that our data do not completely rule out the possibility of PFK contributing to GNG. Based on the qPCR, PFK is depleted to 20 % in the parental cell line (not by), and to 60 % in the double KO background. We have adjusted the title of the paragraph, and extended the Discussion, where we find these speculations more appropriate.*

Fernandes and colleagues [17] showed reverse PFK activity (equal to canonical FBPase) in

vitro. Our data do not support reverse activity of trypanosome PFK in vivo, however, since we

observed a decrease in glycolytic flux but not GNG flux following PFK knockdown.

Please revise this sentence, which is not clear.

*The sentence was revised to be more clear (lines 577 – 585):*

*Fernandes and colleagues [17] showed reverse PFK activity (equal to canonical FBPase) in vitro and proposed that the glycosomal microenvironment might create conditions for reverse PFK activity. However, our data do not support this view because, although PFK RNAi was not complete (decrease in PFK expression to 20 % in parental and to 60 % in the Δfbp.sbp background), we could detect a major decrease in glycolytic flux but not in GNG flux. However, if reactions in both directions occur simultaneously, we would not be able to detect a minor change in GNG flux.*

Fernandes et al. calculated that the reversal should occur if the concentration of substrates is 500-fold higher than the concentration of products [17].

This is not in agreement with the equillibrium constants. It is predicted that the product of both substrates (without considering other factors) should be in the range of 350 at middle-low intracellular pH. Which means that, at equal concentrations of co-products each one is 20 times in excess. Of course, this condition is unlikely, but allow to figure out the existence of such conditions. If the pH is 6.0, the equilibrium constant falls dramatically to 60. Making the same rough estimation, the needed excess of products in relation to substrates is 7.75 times.

*We adjusted our statement to address the reviewer´s comment (lines 590 – 608). It now reads as follows:*

*The directionality of the PFK activity is dependent on concentrations of substrates and products, but the negative free energy (ΔG) of the PFK reaction is highly favorable for the forward reaction. Significant perturbations in substrates concentrations including ADP/ATP ratio and changes in glycosomal pH might allow the PFK reversal [17]. Nevertheless, in our metabolomic datasets we did not show any prominent changes in ADP, ATP, F6P, and F1,6bP levels, for instance one of the largest changes is the 2-fold accumulation of F1,6bP in Δfbp.sbp/^RNAi^PFK compared with WT (S3 Fig). Altogether, our data do not support participation of PFK in GNG. However, it should be noted that we measured metabolites extracted from whole cells, whereas metabolite levels and ADP/ATP ratios may be significantly shifted within the glycosomal subcompartment compared to that of the whole cell. The application of technically challenging methods is required to determine the metabolic microenvironment of glycosomes to gain a deeper understanding of the involvement of individual enzymes in GNG.*
